# Determinants of referral of women with urinary incontinence to specialist services: a national cohort study using primary care data from the UK

**DOI:** 10.1186/s12875-020-01282-y

**Published:** 2020-10-16

**Authors:** Ipek Gurol-Urganci, Rebecca S. Geary, Jil B. Mamza, Masao Iwagami, Dina El-Hamamsy, Jonathan Duckett, Andrew Wilson, Douglas Tincello, Jan van der Meulen

**Affiliations:** 1 Department of Health Services Research and Policy, London School of Hygiene and Tropical Medicine, 15-17 Tavistock Place, London, WC1H 9SH UK; 2 Centre for Quality Improvement and Clinical Audit, Royal College of Obstetricians and Gynaecologists, London, UK; 3 Department of Health Services Research, University of Tsukuba, Tsukuba, Japan; 4 Department of Non-Communicable Disease Epidemiology, London School of Hygiene and Tropical Medicine, London, UK; 5 Leicester General Hospital, Women’s and Children’s Clinical Business Unit, University Hospitals of Leicester NHS Trust, Leicester, UK; 6 Medway NHS Foundation Trust, Gillingham, Kent, UK; 7 Department of Health Sciences, College of Life Sciences, University of Leicester, Leicester, UK

**Keywords:** Female urinary incontinence, Referral, General practice, Primary care

## Abstract

**Background:**

Female urinary incontinence is underdiagnosed and undertreated in primary care. There is little evidence on factors that determine whether women with urinary incontinence are referred to specialist services. This study aimed to investigate characteristics associated with referrals from primary to specialist secondary care for urinary incontinence.

**Methods:**

We carried out a cohort study, using primary care data from over 600 general practices contributing to the Clinical Practice Research Datalink (CPRD) in the United Kingdom. We used multi-level logistic regression to estimate adjusted odds ratios (aOR) that reflect the impact of patient and GP practice-level characteristics on referrals to specialist services in secondary care within 30 days of a urinary incontinence diagnosis. All women aged ≥18 years newly diagnosed with urinary incontinence between 1 April 2004 and 31 March 2013 were included. One-year referral was estimated with death as competing event.

**Results:**

Of the 104,466 included women (median age: 58 years), 28,476 (27.3%) were referred within 30 days. Referral rates decreased with age (aOR 0.34, 95% CI 0.31–0.37, comparing women aged ≥80 with those aged 40–49 years) and was lower among women who were severely obese (aOR 0.84, 95% CI 0.78–0.90), smokers (aOR 0.94, 95% CI 0.90–0.98), women from a minority-ethnic backgrounds (aOR 0.76, 95% CI 0.65–0.89 comparing Asian with white women), women with pelvic organ prolapse (aOR 0.77, 95% CI 0.68–0.87), and women in Scotland (aOR 0.60, 95% CI 0.46–0.78, comparing women in Scotland and England). One-year referral rate was 34.0% and the pattern of associations with patient characteristics was almost the same as for 30-day referrals.

**Conclusions:**

About one in four women with urinary incontinence were referred to specialist secondary care services within one month after a UI diagnosis and one in three within one year. Referral rates decreased with age which confirms concerns that older women with UI are less likely to receive care according to existing clinical guidelines. Referral rates were also lower in women from minority-ethnic backgrounds. These finding may reflect clinicians’ beliefs about the appropriateness of referral, differences in women’s preferences for treatment, or other factors leading to inequities in referral for urinary incontinence.

## Background

Urinary incontinence (UI), involuntary loss of urine, affects 50% of women at some point in their lives and it has a substantial impact on their quality of life [1–5]. Prevalence increases with age ranging from 17% in women aged over 20 years to 38% in those older than 60 years [6–8]. Only 25% of women with UI seek care, and of those, less than half receive treatment suggesting a high unmet need for care [9–11]. Delays in seeking treatment can amount to several years [12]. When left untreated, incontinence is associated with falls and fractures, depression, and sleep disturbance [13–15].

General practitioners (GPs) are the gatekeepers of healthcare in the UK National Health Service (NHS), acting as the first point-of-contact for most non-emergency health issues in primary care. In the UK, UI is expected to be initially managed at primary-care level [11]. Most women do not require extensive preliminary evaluations because first-line non-invasive treatments, including pelvic floor exercises and lifestyle changes, may be begin without clear differentiation of the sub-type of UI (e.g. stress, urgency or mixed incontinence) [16]. Referral to an incontinence specialist is recommended when these first-line treatments do not sufficiently improve symptoms or are not acceptable to women. Urgent referral is only recommended if there are concerns about conditions such as cancer or persistent pain [16, 17].

Decisions regarding referral and further treatment for UI will depend on a wide range of factors, including the severity of UI, its impact on daily activities, a woman’s goals and expectations for improvement or cure and her acceptance of the adverse effects of conservative and more invasive treatments [5]. There is some evidence suggesting that some women, such as older women among whom UI is most prevalent, are less likely to receive continence care in line with national clinical guidelines [18]. We used electronic data derived from primary care practices in the UK to study the extent to which referral to secondary care is associated with the characteristics of the women.

## Methods

### Study design and setting

We conducted a national cohort study using data from the Clinical Practice Research Datalink (CPRD), which is the largest validated computerised database of anonymised longitudinal medical records for primary care in the world. CPRD contains data on demographic and lifestyle factors, clinical events (symptoms, diagnoses, tests, referrals) from over 10.5 million patient records from over 600 practices, covering approximately 6.9% of the UK population [19]. It is representative of the general population in terms of age, sex and ethnicity, and comparable for body mass index (BMI) distribution to the Health Survey for England [19]. Primary care practices contributing to the CPRD need to meet prespecified data entry quality criteria defined by CPRD as ‘up-to-standard for use in research’ [19].

### Study population

We identified all women aged 18 years or older who had an ‘index’ diagnosis of UI (defined using the Read codes in Supplementary Table S1) between 1 April 2004 and 31 March 2013. An index diagnosis was defined as a diagnosis of UI among women who had no earlier record of a UI symptom/diagnosis (Supplementary Table S2) or treatment (Supplementary Table S3) within the 12 months prior to the date of first diagnosis in the study period. To adequately account for patient history, women with less than 12 months of up-to-standard data prior to index diagnosis were excluded. Women were followed up from the index diagnosis until the end of follow-up (earliest of the date of a referral to a UI specialist, transfer out of practice, death or 1 April 2014). Women were also excluded if the follow-up period was less than 30 days.

### Outcome

The primary outcome was any referral to a UI specialist within 30 days of a urinary incontinence diagnosis. Referral to a specialist was defined using a combination of Read Codes and referral specialty codes (Supplementary Table S4). In an additional analysis, we also investigated referral within 1 year. For this additional analysis, women were excluded if the follow-up period was less than 1 year.

### Patient and practice-level characteristics

Patient and practice-level characteristics were selected apriori on the basis of clinical significance. Patient characteristics were: age at index UI diagnosis (18–39, 40–49, 50–59, 60–69, 70–79, ≥80 years), BMI (kg/m^2^; < 20 = underweight, 20–24 = normal, 25–29 = overweight, 30–39 = obese, ≥40 = severely obese), smoking status (non, current smoker, or ex smoker) and ethnic background (white, Asian/Asian-British, black/black-British, mixed or other ethnic group, and missing). Comorbidities included were pelvic organ prolapse, urinary tract infection (UTI), type 2 diabetes mellitus, cardiovascular disease: defined as a diagnosis of any cardiovascular or ischaemic heart disease, heart failure or hypertension), renal disease, respiratory disease (asthma or chronic obstructive pulmonary disease, anxiety or depression, and cancer. Comorbidities were defined from the presence or absence of relevant Read codes within the 12 months before the index UI diagnosis date, except for UTI which was identified in the 30 days before the index date. BMI and smoking status were defined using the value recorded closest to the index date. No time-restrictions were placed on codes recorded for ethnicity. The code lists to identify comorbidities were obtained from the clinical codes repository with the exception of pelvic organ prolapse, which was developed by the research team through computerised search and manual review of codes by clinical experts (Supplementary Table S5) [20].

Practice-level characteristics used were practice Index of Multiple Deprivation score (an area-level measure of economic deprivation determined based on general practice postcode) analysed as quintiles of the national distribution ranging from 1 (most deprived) to 5 (least deprived) and country (England, Northern Ireland, Scotland and Wales) [19].

### Statistical analyses

We used proportions and 95% confidence intervals (95% CIs) to summarise patient and practice characteristics. For the analysis of the 1-year rate of referral, we used the cumulative incidence function. Patients reaching the end of the follow-up period were censored. Death was considered as a competing event [21].

Multi-level multivariable logistic regression with was conducted to identify patient and practice-level characteristics associated with referral within 30 days, expressed as odds ratios (ORs) with 95% CIs. Wald tests were used to test whether the association between patient/practice-level characteristics and referral were statistically significant.

Levels of missing values were generally low (< 5% for all characteristics apart from ethnicity). Multiple imputation using chained equations was used to impute missing values for BMI (missing for 5% of women) and smoking status (missing for 0.1% of women) with statistical coefficients obtained from pooling results over 10 imputed data sets using Rubin’s rules [22]. Ethnicity data were missing for 55% of women, a level of missingness too high to use multiple imputation. As a result, we included a separate ‘missing’ category for ethnicity in the regression models. Analyses were performed using Stata version 15 [23].

## Results

### Patient characteristics

**Fig. 1:**
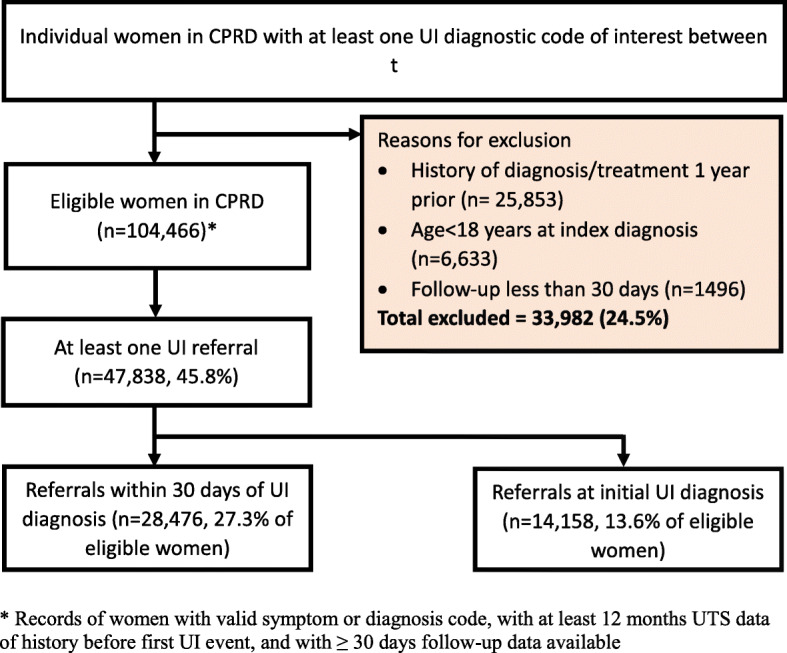
Selection of 104,466 patients with initial diagnosis of urinary incontinence from CPRD. * Records of women with valid symptom or diagnosis code, with at least 12 months UTS data of history before first UI event, and with ≥30 days follow-up data available

Between April 2004 and March 2014, 138,448 women had an index diagnosis of UI, of whom 104,466 met the inclusion criteria (Fig. 1). The median age of women in the cohort was 58 years. About two thirds of women were overweight (32.1%) obese (29.1%), or severely obese (8.2%) (Table 1). Of the women with available ethnicity data, 92.4% were white, 3.7% Asian/Asian British and 2.0% black/black British. Of the comorbidities considered, cardiovascular disease and anxiety or depression were the most common, each recorded for approximately 12% of women.

**Table 1:** Patient characteristics associated with referral within 30 days to specialist continence services in secondary care

	Total (%)	Referred (%)	Rate of referral	Adjusted odds ratio 95%CI	***P*** value
**Overall**	104,466	28,476	27.3%		
Age (median 58, IQR 45–73)					
Age group (years)					
18–39	14,599 (14)	4696 (16.5)	32.2%	0.91 (0.87, 0.96)	
40–49	21,642 (20.7)	7411 (26.0)	34.2%	Reference	< 0.001
50–59	19,654 (18.8)	5964 (20.9)	30.3%	0.84 (0.80, 0.88)	
60–69	17,468 (16.7)	4687 (16.5)	26.8%	0.70 (0.66, 0.73)	
70–79	15,834 (15.2)	3372 (11.8)	21.3%	0.51 (0.49, 0.54)	
≥ 80	15,269 (14.6)	2346 (8.2)	15.4%	0.34 (0.31, 0.37)	
BMI (kg/m^2^)					
Underweight (< 20)	5224 (5.3)	1190 (4.4)	22.8%	0.85 (0.79, 0.91)	
Normal (20–24)	28,044 (28.3)	7966 (29.2)	28.4%	Reference	< 0.001
Overweight (25–29)	31,580 (31.8)	8748 (32.1)	27.7%	0.99 (0.95, 1.03)	
Obese (30–39)	28,873 (29.1)	7922 (29.1)	27.4%	0.95 (0.91, 0.99)	
Severely obese (≥40)	5474 (5.5)	1439 (5.3)	26.3%	0.84 (0.78, 0.90)	
Smoking status					
Non-smoker	61,109 (58.6)	16,471 (57.9)	27.0%	Reference	< 0.001
Current	18,827 (18)	5350 (18.8)	28.4%	0.94 (0.90, 0.98)	
Ex-smoker	24,395 (23.4)	6632 (23.3)	27.2%	1.04 (1.01, 1.08)	
Ethnicity					
White	43,015 (92.4)	11,398 (92.9)	26.5%	Reference	0.001
Asian/Asian British	1722 (3.7)	416 (3.4)	24.2%	0.76 (0.65, 0.89)	
Black/Black British	930 (2)	221 (1.8)	23.8%	0.76 (0.62, 0.92)	
Mixed/Other	888 (1.9)	233 (1.9)	26.2%	0.85 (0.69, 1.05)	
Missing (55.4%)	–	–	28.0%	1.04 (0.97, 1.11)	
Comorbidities					
Urinary tract infection	2503 (2.4)	659 (2.3)	26.3%	1.10 (1.00, 1.21)	0.06
Pelvic organ prolapse	3230 (3.1)	720 (2.5)	22.3%	0.77 (0.68, 0.87)	0.00
Type 2 diabetes mellitus	5639 (5.4)	1221 (4.3)	21.7%	0.92 (0.85, 0.99)	0.02
Cardiovascular disease^5^	12,034 (11.5)	2632 (9.2)	21.9%	0.95 (0.90, 1.00)	0.07
Renal disease	2507 (2.4)	491 (1.7)	19.6%	0.97 (0.86, 1.09)	0.59
Respiratory disease	9396 (9)	2590 (9.1)	27.6%	1.01 (0.96, 1.06)	0.68
Anxiety or depression	12,101 (11.6)	3358 (11.8)	27.7%	0.95 (0.90, 1.00)	0.05
Cancer	1785 (1.7)	365 (1.3)	27.3%	0.84 (0.75, 0.94)	0.00

### Referral to a continence specialist

Of the 104,466 women with UI, 47,838 had a referral to a UI specialist in secondary care at some point during the study period (45.8%, Fig. 1). Of these, 14,158 women (13.6, 95% CI 13.3–13.8%) had a referral recorded on the same day as the index UI diagnosis. The cumulative incidence of referral with death as a competing event was 25.5% (95% CI 25.3–25.8%) at 30 days and 34.0%, (95% CI 33.7–34.3%) at one year.

**Fig. 2:**
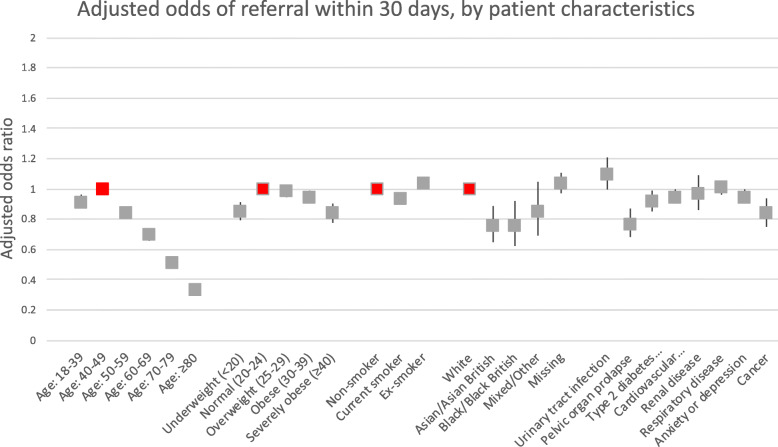
Adjusted odds (with 95% confidence intervals) of referral by patient characteristics. See also Table 1. Red symbols indicate the reference category

The rate of referral rate within 30 days of the recording of the index UI diagnosis was highest in women aged between 40 and 49 years 34.3% (Table 1). Figure 2 shows that the 30-day referral rate rapidly decreased with age to 15.4% in women aged 80 years or above (adjusted OR compared to women aged between 40 and 49 0.34, 95% CI 0.31–0.37). Women from minority-ethnic background were less often referred than white women (adjusted ORs compared to white women 0.76, 95% CI 0.65–0.89, for Asian women and 0.76, 95% CI 0.62–0.92, for black women).

Women who were underweight (adjusted OR compared to normal weight 0.85, 95% CI 0.79–0.91) and those who were severely obese (adjusted OR 0.84, 95% CI 0.78–0.90) were less often referred than women with a BMI in the normal range. Women who were current smokers were less often referred than non-smokers (adjusted OR 0.94, 95% CI 0.90–0.98), just as women with a diagnosis of pelvic organ prolapse (adjusted OR 0.77, 95% CI 0.68–0.87), women with type 2 diabetes (adjusted OR 0.92, 95% CI 0.85–0.99) and women diagnosed with cancer (adjusted OR 0.84, 95% CI 0.75–0.94). Other comorbidities were not associated with referral.

The country in which the primary care practice that recorded the index UI diagnosis was located was also associated with 30-day rate of referral (Table 2). Referral rates were lower in Scotland (adjusted OR 0.60 compared to England, 95% CI 0.46–0.78) and higher in Northern Ireland (adjusted OR 1.83, 95% CI 1.40–2.39). The referral rate was not associated with the the level of socioeconomic deprivation of the area in which the primary care practice was located.

**Table 2:** Primary care practice-level characteristics associated with referral within 30 days to specialist continence services in secondary care

	Total (%)	Referred (%)	Rate of referral	Adjusted odds ratio 95%CI	P value
**Overall**	104,466	28,476	27.3%		
Country					
England	80,751 (77.3)	22,189 (77.9)	27.5%	Reference	< 0.001
Northern Ireland	4187 (4.0)	1774 (6.2)	42.4%	1.83 (1.40, 2.39)	
Scotland	10,908 (10.4)	2049 (7.2)	18.8%	0.60 (0.46, 0.78)	
Wales	8620 (8.3)	2464 (8.7)	28.6%	1.05 (0.89, 1.24)	
Seocio-economic deprivation (national quintiles)					
1 Most deprived	19,485 (18.7)	5486 (19.3)	28.2%	Reference	0.16
2	20,782 (19.9)	6024 (21.2)	29.0%	1.02 (0.88, 1.19)	
3	20,576 (19.7)	5706 (20)	27.7%	1.00 (0.86, 1.16)	
4	21,960 (21)	5750 (20.2)	26.2%	0.90 (0.77, 1.04)	
5 Least deprived	21,663 (20.7)	5510 (19.3)	25.4%	0.88 (0.74, 1.05)	

As explained in the Methods section, we carried out an additional analysis of referral within one year and found that the pattern of associations between patient characteristics and referral, expressed in terms of adjusted ORs, was almost the same as reported for referral within 30 days.

## Discussion

### Summary

About a quarter of women newly diagnosed with UI in primary care in the UK between April 2004 and March 2013 were referred to specialist services in secondary within 30 days and about a third were referred within one year. Age and ethnicity were strongly associated with referral. Older women and those from minority-ethnic backgrounds were much less likely to be referred to a specialist than younger women and white women. Other factors strongly associated with referral were the additional presence of pelvic organ prolapse, which reduced the referral rate, and the country in which primary care was sought.

### Strengths and weaknesses

The main strength of the study was the large cohort of women with UI. The CPRD, the primary care database used for this study, is a representative sample of primary care patients in the UK and therefore, our findings can be considered generalisable to the national population of women with UI who visit their GP [19, 24]. The overall quality of clinical information in CPRD has been found to be sufficiently high for research purposes [19] and our analysis was restricted to general practices considered ‘up-to-standard for use in research’ by CPRD during the study period.

The analysis was subject to several weaknesses inherent to database studies, where the level of detail may be limited [19]. First, we did not have information about the severity of UI and its impact on quality-of-life. As a result, we were unable to account for potential variation in the average severity of UI between groups of women which may have contributed to some of the observed variation in referral rates between specific groups of women. Neither did we have information about the impact of the incontinence on the women’s daily life or the level of discomfort. As a result, we cannot distinguish whether the identified referrals were motivated by a desire for treatment of the urinary incontinence or concerns about possible underlying health problems.

A second weakness of conducting research using administrative healthcare data is that the absence of a code for a condition must be interpreted as absence of the condition. Potential misclassification may therefore arise from variation and inconsistencies in coding diagnoses by the GPs [25]. To minimise the impact of this we used published code lists as far as possible. Where published lists could not be identified, we developed these by combining a thorough computerised search of codes with review by clinical experts, and publish them as supplementary material for future research.

Third, a specific weakness of the CPRD is the high proportion of patients for whom ethnicity data were missing which undermines its value for research into inequalities. To overcome this issue, we used a separate ‘missing’ category for ethnicity The consequence of this approach is that the adjusted results for the other variables in the multivariable model are only partially adjusted for differences in the women’s ethnic background as they reflect the weighted average of two associations: the association between referral adjusted for ethnicity among the women for whom data on their ethnic background was available and the association between referral unadjusted for ethnicity among women for whom data on their ethnic background was missing. Given that the level of missingness was very similar in women who were and were not referred and the fact that more than 90% of women whose ethnic background was known had a white ethnic background, it is very unlikely that the use of a separate missing category had a large impact on our results [26].

Fourth, historical measurements, such as for BMI, may not always be appropriate indicators of a patient’s risk later in follow-up [27]. However, this is unlikely to have had a major effect in our study as the follow-up period for the primary outcome was short (between 30 days and 1 year).

Lastly, we could only include patients whose UI was first recorded between 2004 and 2014. However, there have not been major changes in the initial management of UI in primary care, including the detection and treatment of modifiable risk factors for UI [16].

### Interpretation of findings

A recent national audit carried out in NHS hospitals in the United Kingdom showed that older women with UI were less likely to have a continence history taken and to receive care according to national guidelines [18]. This evidence is in line with our findings that older women had lower rates of referral from primary care to a continence specialist in secondary care.

Specific clinical considerations will have influenced whether or not women with UI are referred to specialist services in secondary care. For example, we found that referral rates were lower in women with UI who smoked or in those who were obese. This may reflect that some GPs may want to explore to what extent lifestyle changes would reduce the severity of the UI. Also, women with a pelvic organ prolapse were less likely to have been referred to an incontinence specialist than women without a prolapse. One explanation could be that women with a prolapse had already been referred in the past [17]. The lower rate of referral in older women may reflect that GPs first try conservative treatment, possibly given the higher levels of frailty not captured by the comorbidities recorded in our data.

We also observed substantial differences in the referral rates according to country. Compared to women in England, women with UI in Scotland were less often and women in Northern Ireland were more often referred (both compared to England). These differences demonstrate the impact of the different healthcare systems across the UK.

### Implications for practice and research

We found that referral rates to specialist continence services in secondary care varied substantially for different groups of women, especially women who are older or from a non-white ethnic background. Research is required to determine to what extent this variation in referral rate reflects differences in clinical need and patient choice on the one hand or inequities in referral for specialist incontinence care on the other.

The research should focus on the clinical assessment of individual pateints with UI in primary care (including history taking and physical examination as well as scoring of symptom severity and quality of life assessment), the availability of conservative treatments (including lifestyle interventions and pelvic floor muscle training), and how women are being supported in making decisions about their care [17].

Also, the delivery and organisation of continence services in primary and secondary care should be scrutinised [28]. Clear referral pathways and investment in capacity, for example, through the provision of more trained staff and a higher profile for continence care within medical training, have been identified as possible facilitators for the delivery of high-quality and equitable continence services.

## Conclusions

About one in four women with urinary incontinence were referred to specialist secondary care services within one month after a UI diagnosis and one in three within one year. Older women and those from minority-ethnic backgrounds were much less likely to be referred to a specialist than younger women and white women. These findings may reflect variations in clinicians’ beliefs about the appropriateness of referral, differences in women’s preferences for treatment, or other factors leading to inequities in referral for urinary incontinence.

## Supplementary information

**Additional file 1.**

## Data Availability

The data analysed is this study is available from the CPRD, a real-world research service supporting retrospective and prospective public health and clinical studies (www.cprd.com). CPRD is jointly sponsored by the Medicines and Healthcare products Regulatory Agency and the National Institute for Health Research (NIHR), as part of the English Department of Health and Social Care.

## Abbreviations

aOR: adjusted odds ratio
BMI: body mass index
CI: confidence interval
CPRD: Clinical Practice Research Datalink
GP: General practitioner
NHS: National Health Service
OR: odds ratio
UI: Urinary incontinence
UTI: Urinary tract infection
