# Supplementary material for: Determinants of referral of women with urinary incontinence to specialist services: a national cohort study using primary care data from the UK
Source: BMC Fam Pract. 2020 Oct 16;21:211. doi: 10.1186/s12875-020-01282-y (PMC7568393; doi:10.1186/s12875-020-01282-y)
Supplement: Supplementary file 1 — Additional file 1. [file 12875_2020_1282_MOESM1_ESM.docx]

### Supplementary Material

### Determinants of referral of women with urinary incontinence to specialist services: a national cohort study using primary care data from the UK

Ipek Gurol-Urganci,^1,2^* Rebecca S Geary,^1,2^* Jil B Mamza,^1^ Masao Iwagami,^3,4^ Dina El-Hamamsy,^5^ Jonathan Duckett,^6^ Andrew Wilson,^7^ Douglas Tincello,^5,7^ Jan van der Meulen^1,2^

| Supplementary Table S1:Read codes to identify urinary incontinence symptoms / diagnoses (CPRD data) | Page 2 |
| --- | --- |
| Supplementary Table S2:Read codes to identify history of urinary incontinence procedures / treatments (CPRD data) | Page 3 |
| Supplementary Table S3:Read codes to identify referrals to secondary care (CPRD Referrals data) | Page 6 |
| Supplementary Table S4:Identification of referrals for urinary incontinence to secondary care (CPRD Referrals data) | Page 11 |
| Supplementary Table S5:Identification of comorbidities (CPRD data) | Page 12 |

### Supplementary Table S1: Read codes to identify urinary incontinence symptoms / diagnoses (CPRD data)

| **Read Code category** | **Read Code** | **CPRD MedCODE** | **Read Code Description** |
| --- | --- | --- | --- |
| 1 - history & symptoms | 1593. | 15918 | H/O: stress incontinence |
| 1 - history & symptoms | 1A23.00 | 6161 | Incontinence of urine |
| 1 - history & symptoms | 1A23000 | 110001 | Functional urinary incontinence |
| 1 - history & symptoms | 1A24.00 | 1929 | Stress incontinence |
| 1 - history & symptoms | 1A24.11 | 5844 | Stress incontinence - symptom |
| 1 - history & symptoms | 1A26.00 | 3887 | Urge incontinence of urine |
| K - genitourinary system diseases | K198.00 | 3182 | Stress incontinence |
| K - genitourinary system diseases | K586.00 | 17620 | Stress incontinence - female |
| K - genitourinary system diseases | Kyu5A00 | 52763 | Other specified urinary incontinence |
| R - symptoms, signs & ill-defined conditions | R083.00 | 3283 | Incontinence of urine |
| R - symptoms, signs & ill-defined conditions | R083000 | 4375 | Enuresis NOS |
| R - symptoms, signs & ill-defined conditions | R083100 | 31220 | Urethral sphincter incontinence |
| R - symptoms, signs & ill-defined conditions | R083200 | 17320 | Urge incontinence |
| R - symptoms, signs & ill-defined conditions | R083z00 | 15400 | Incontinence of urine NOS |

### Supplementary Table S2: Read codes to identify history of urinary incontinence procedures / treatments (CPRD data)

| **Read Code Chapter** | **Read Code** | **CPRD MedCODE** | **Read Code Description** |
| --- | --- | --- | --- |
| 3 - diagnostic procedures | 3940 | 13421 | Bladder: incontinent |
| 3 - diagnostic procedures | 3941 | 13422 | Bladder: occasional accident |
| 7 - operations, procedures & sites | 7B3..11 | 2498 | Bladder neck operations |
| 7 - operations, procedures & sites | 7B30.00 | 40515 | Combin abdominal & vaginal ops support outlet female bladder |
| 7 - operations, procedures & sites | 7B30000 | 26236 | Abdominoperineal suspension of urethra |
| 7 - operations, procedures & sites | 7B30100 | 34857 | Unspecified endoscopic suspension of bladder neck |
| 7 - operations, procedures & sites | 7B30200 | 36712 | Stamey endoscopic bladder neck suspension |
| 7 - operations, procedures & sites | 7B30300 | 67945 | Pereyra - Raz endoscopic bladder neck suspension |
| 7 - operations, procedures & sites | 7B30400 | 29989 | Gittes endoscopic bladder neck suspension |
| 7 - operations, procedures & sites | 7B30y00 | 35658 | Combined abdo & vaginal op to support outlet fem bladder OS |
| 7 - operations, procedures & sites | 7B30z00 | 35662 | Combined abdo & vaginal op to support outlet fem bladder NOS |
| 7 - operations, procedures & sites | 7B31000 | 15696 | Suprapubic sling operation |
| 7 - operations, procedures & sites | 7B31011 | 21250 | Aldridge suprapubic sling |
| 7 - operations, procedures & sites | 7B31014 | 64217 | Suprapubic urethrovesical suspension |
| 7 - operations, procedures & sites | 7B31100 | 12737 | Retropubic suspension of bladder neck |
| 7 - operations, procedures & sites | 7B31112 | 4313 | Marshall- Marchetti suspension |
| 7 - operations, procedures & sites | 7B31113 | 34104 | Marshall-Marchetti-Krantz retropubic suspension of urethra |
| 7 - operations, procedures & sites | 7B31200 | 4202 | Colposuspension of bladder neck |
| 7 - operations, procedures & sites | 7B31211 | 17771 | Burch colposuspension |
| 7 - operations, procedures & sites | 7B31z00 | 71003 | Abdominal operation to support outlet of female bladder NOS |
| 7 - operations, procedures & sites | 7B32000 | 31461 | Vaginal buttressing of urethra |
| 7 - operations, procedures & sites | 7B32011 | 45544 | Kelly urethrovesical plication |
| 7 - operations, procedures & sites | 7B32012 | 98338 | Kennedy urethrovesical plication |
| 7 - operations, procedures & sites | 7B32200 | 11197 | Introduction of tension free vaginal tape |
| 7 - operations, procedures & sites | 7B32500 | 57283 | Introduction of transobturator tape |
| 7 - operations, procedures & sites | 7B32y00 | 49097 | Vaginal operation to support outlet of female bladder OS |
| 7 - operations, procedures & sites | 7B32z00 | 48900 | Vaginal operation to support outlet of female bladder NOS |
| 7 - operations, procedures & sites | 7B33400 | 46819 | Insertion of sphincter around female bladder neck |
| 7 - operations, procedures & sites | 7B33411 | 42425 | Implantation of sphincter around female bladder neck |
| 7 - operations, procedures & sites | 7B33412 | 52000 | Insertion artificial urinary sphincter in outlet fem bladder |
| 7 - operations, procedures & sites | 7B33600 | 49520 | Maintenance of bladder neck sphincter in female |
| 7 - operations, procedures & sites | 7B33800 | 98767 | Insertion retropubic device stress urinary incontinence NEC |
| 7 - operations, procedures & sites | 7B33B00 | 89908 | Reconstruction of neck of female bladder NEC |
| 7 - operations, procedures & sites | 7B34200 | 36539 | Endoscopic suburethral injection of inert substance - female |
| 7 - operations, procedures & sites | 7B34211 | 18434 | Endoscopic suburethral injection of collagen in female |
| 7 - operations, procedures & sites | 7B34212 | 46542 | Endoscopic suburethral teflon injection in female |
| 7 - operations, procedures & sites | 7B34300 | 40010 | Endoscopic uroplastique injection outlet of female bladder |
| 7 - operations, procedures & sites | 7B38900 | 97037 | Introduction of transobturator sling |
| 8 - other therapeutic procedures | 8C14.00 | 2739 | Incontinence care |
| 8 - other therapeutic procedures | 8D7..12 | 17637 | Incontinence control |
| 8 - other therapeutic procedures | 8D71.00 | 48601 | Incontinence control |
| Z - unspecified conditions | Z9EA.00 | 45495 | Provision of incontinence appliance |

### Supplementary Table S3: Read codes to identify referrals to secondary care (CPRD Referrals data)

| **CPRD MedCODE** | **Read Code** | **Read Code description** |
| --- | --- | --- |
| Physiotherapy* | | |
| 2407 | 8E...00 | Physiotherapy/remedial therapy |
| 213 | 8E...11 | Physiotherapy |
| 24338 | 8EZ..00 | Other physiotherapy |
| 69779 | 8E74.11 | Pelvic floor exercises |
| 17237 | 8E77.00 | Pelvic floor exercises |
| 9020 | 8E97.00 | Bladder training |
| 33268 | 8E97000 | Bladder drill |
| 8437 | 9NJ3.00 | In-house physio |
| 6010 | 9NJ4.00 | In-house physiotherapy - domiciliary visit |
| 10089 | ZL85.00 | Referral to physiotherapist |
| 8164 | ZL85.11 | Refer to physiotherapist |
| 11894 | ZL85100 | Referral to community-based physiotherapist |
| 13681 | ZL85111 | Referral to community physiotherapist |
| 32769 | ZL85200 | Referral to hospital-based physiotherapist |
| 12282 | ZL85211 | Referral to hospital physiotherapist |
| 8543 | 8HH5.00 | Refer to domiciliary physiotherapy |
| 31147 | 8HHA.00 | Refer to community physiotherapist |
| 1116 | 8H77.00 | Refer to physiotherapist |
| 13671 | 8HVb.00 | Private referral to physiotherapist |
|  |  |  |
| Continence Care/Assessment (including urodynamics) | | |
| 7650 | 394..00 | Bladder - assessment |
| 13424 | 394..11 | Bladder-incontinence assessmnt |
| 13423 | 394..12 | Bladder- continence assessment |
| 13421 | 3940 | Bladder: incontinent |
| 13422 | 3941 | Bladder: occasional accident |
| 13420 | 3942 | Bladder: fully continent |
| 12424 | ZQ3H.00 | Bladder assessment |
| 40789 | 39H..00 | Continence assessment |
| 49417 | 39H0.00 | Continence reassessment |
|  |  |  |
| 5269 | 317..00 | Special urinary procedures |
| 15290 | 317..11 | Urinary - special tests |
| 2916 | 317..12 | Urodynamic studies |
| 20140 | 3174 | Special urinary test abnormal |
| 41472 | 3174.11 | Urodynamic studies abnormal |
| 40731 | 3174000 | Cystometry abnormal |
| 12169 | 3175 | Detrusor reflex testing |
| 10876 | 3176 | Residual urinary volume |
| 103500 | 3177 | Uroflowmetry |
| 103851 | 3178 | Voided urinary volume |
| 103674 | 3179 | Average urinary flow rate |
| 20728 | 317A.00 | Pad test for incontinence |
| 6716 | 317B.00 | Other urodynamic tests |
| 18036 | 317C.00 | Urinary flow rate |
| 104865 | 317D.00 | Time to maximum urinary flow |
| 105951 | 317D.11 | TQmax - Time to maximum urinary flow rate |
| 103615 | 317E.00 | Urinary voiding total flow time |
| 103913 | 317F.00 | Urinary flow time |
| 14962 | 317Z.00 | Special urinary procedure NOS |
| 64138 | 7P14300 | Urodynamics NEC |
|  |  |  |
| 2739 | 8C14.00 | Incontinence care |
| 12138 | 8C14.11 | Continence care |
| 22095 | ZLA2400 | Seen by continence nurse |
|  |  |  |
| 18998 | 8HR6.00 | Refer to Urodynamic studies |
| 29192 | 8H7w.00 | Referral to continence nurse |
| 25899 | 8HTX.00 | Referral to incontinence clinic |
| 25901 | ZL62400 | Referral to continence nurse |
|  |  |  |
| Gynaecology/Urology/GUM referral | | |
| 48014 | 8H4V.00 | Referral to gynaecology special interest GP |
| 2116 | 8H58.00 | Gynaecological referral |
| 103854 | 8Hku.00 | Referral to community gynaecology service |
| 31873 | 8HMO.00 | Listed for Gynae admission |
| 13647 | 8HV7.00 | Private referral to gynaecologist |
| 9966 | ZL5D.00 | Referral to obstetrician and gynaecologist |
| 10663 | ZL5D200 | Referral to gynaecologist |
|  |  |  |
| 6589 | 8H4A.11 | Referred to genito urinary physician |
| 30868 | 8H4W.00 | Referral to urology special interest general practitioner |
| 2568 | 8H5B.00 | Referred to urologist |
| 13704 | 8HTa.00 | Referral to genitourinary clinic |
| 13644 | 8HVA.00 | Private referral to urologist |
| 23104 | ZL5AJ00 | Referral to genitourinary physician |
| 10313 | ZL5GP00 | Referral to urologist |
|  |  |  |
| Further care/general medicine/general surgeon referral* | | |
| 91 | 8H...00 | Referral for further care |
| 13674 | 8H4..00 | Referral to physician |
| 1861 | 8H4..11 | Medical referral |
| 11219 | 8H4..12 | Refer to physician |
| 7124 | 8H41.00 | General medical referral |
| 20251 | 8H4Z.00 | Referral to physician NOS |
| 43014 | ZL5A.00 | Referral to physician |
| 10449 | ZL5AE00 | Referral to general physician |
|  |  |  |
| 22670 | 8H5..00 | Referral to surgeon |
| 3016 | 8H5..11 | Surgical referral |
| 5156 | 8H51.00 | General surgical referral |
| 21020 | 8H5Z.00 | Referral to surgeon NOS |
| 10214 | ZL5G500 | Referral to general surgeon |
|  |  |  |
| 15812 | 8H7..00 | Other referral |
| 6535 | 8H72.00 | Refer to district nurse |
| 3975 | 8H7a.00 | Refer to hospital |
| 2558 | 8HD..00 | Refer to hospital OPD |
| 32882 | 8He..00 | Referral to intermediate care |
| 39479 | 8HH..00 | Referred - other care |
| 19171 | 8HT..00 | Referral to clinic |
| 30263 | ZL6..00 | Referral to nurse |
| 25924 | ZL62.00 | Referral to clinical nurse specialist |
| 11495 | ZL63211 | Refer to district nurse |
| 56102 | ZL65.00 | Referral to nurse practitioner |
|  |  |  |
| 20965 | 8H61.00 | Referral to private doctor |
| 7820 | 8H61.11 | Private referral |
| 17946 | 8HV..00 | Private referral |
| 13634 | 8HV0.00 | Private referral to general surgeon |

(*categories need to be supplemented with a UI diagnosis/symptom code on the day of the referral)

### Supplementary Table S4: Identification of referrals for urinary incontinence to secondary care (CPRD Referrals data)

| **Physiotherapy referral - specialty codes*** | | |
| --- | --- | --- |
| 77 | Physiotherapy | NHSSPEC |
|  |  |  |
| **Gynaecology/Urology/GUM referral - specialty codes** | | |
| 2 | Urology | NHSSPEC |
| 32 | Genito-Urinary Medicine | NHSSPEC |
| 44 | Gynaecology | NHSSPEC |
| 81 | Obstetrics & Gynaecology | NHSSPEC |
|  |  |  |
| 6 | Gynaecology | FSHASPEC |
| 14 | Genito-Urinary | FSHASPEC |
|  |  |  |
| **Further care referral - specialty codes*** | | |
| 1 | General Surgery | NHSSPEC |
| 16 | General Medicine | NHSSPEC |
| 1 | General Surgical | FSHASPEC |
| 2 | General Medical | FSHASPEC |

(*categories need to be supplemented with a UI diagnosis/symptom code on the day of the referral)

### Supplementary Table S5: Identification of comorbidities (CPRD data)

All comorbidities were captured using Read code repositories reported at <https://clinicalcodes.rss.mhs.man.ac.uk/medcodes/articles/> with the exception of those listed below.

| **Method** | |
| --- | --- |
| Pelvic organ prolapse | Keyword searches of Read code directories, reviewed and finalised by clinical advisory team |
| Asthma | Keyword searches of Read code directories, reviewed and finalised by clinical advisory team |
| Cancer | Repository & keyword searches of Read code directories, reviewed and finalised by project team |

| **Pelvic Organ Prolapse** | | | |
| --- | --- | --- | --- |
| **Read Code Chapter** | **Read Code** | **CPRD MedCODE** | **Read Code description** |
| 7 - operations, procedures & sites | 7D19.00 | 17020 | Repair of vault of vagina |
| 7 - operations, procedures & sites | 7D19000 | 66379 | Repair vaginal vault combined abdominal & vaginal approach |
| 7 - operations, procedures & sites | 7D19100 | 52417 | Repair of vault of vagina using abdominal approach NEC |
| 7 - operations, procedures & sites | 7D19200 | 57237 | Repair of vault of vagina using vaginal approach NEC |
| 7 - operations, procedures & sites | 7D19300 | 16175 | Sacrocolpopexy |
| 7 - operations, procedures & sites | 7D19400 | 1652 | Suspension of vagina NEC |
| 7 - operations, procedures & sites | 7D19500 | 18931 | Sacrospinous fixation of vaginal vault |
| 7 - operations, procedures & sites | 7D19600 | 96345 | Repair of vault of vagina with mesh using abdominal approach |
| 7 - operations, procedures & sites | 7D19700 | 46339 | Repair of vault of vagina with mesh using vaginal approach |
| 7 - operations, procedures & sites | 7D19y00 | 27604 | Other specified repair of vault of vagina |
| 7 - operations, procedures & sites | 7D19z00 | 48218 | Repair of vault of vagina NOS |
| K - genitourinary system diseases | K195.11 | 16981 | Urethrocele |
| K - genitourinary system diseases | K51..00 | 6819 | Genital prolapse |
| K - genitourinary system diseases | K510000 | 211 | Cystocele without uterine prolapse |
| K - genitourinary system diseases | K510100 | 25278 | Cystourethrocele without uterine prolapse |
| K - genitourinary system diseases | K510200 | 2285 | Rectocele without uterine prolapse |
| K - genitourinary system diseases | K510211 | 37918 | Proctocele without uterine prolapse |
| K - genitourinary system diseases | K510300 | 4575 | Urethrocele without uterine prolapse |
| K - genitourinary system diseases | K512.00 | 7870 | Uterovaginal prolapse, incomplete |
| K - genitourinary system diseases | K512000 | 30419 | Cystocele with first degree uterine prolapse |
| K - genitourinary system diseases | K512100 | 12359 | Cystocele with second degree uterine prolapse |
| K - genitourinary system diseases | K513.00 | 9356 | Uterovaginal prolapse, complete |
| K - genitourinary system diseases | K513000 | 25974 | Cystocele with third degree uterine prolapse |
| K - genitourinary system diseases | K514.00 | 1057 | Uterovaginal prolapse, unspecified |
| K - genitourinary system diseases | K514000 | 12845 | Cystocele with unspecified uterine prolapse |
| K - genitourinary system diseases | K515.00 | 10888 | Post hysterectomy vaginal vault prolapse |
| K - genitourinary system diseases | K516.00 | 2846 | Vaginal enterocele |
| K - genitourinary system diseases | K516100 | 41136 | Acquired vaginal enterocele |
| K - genitourinary system diseases | K516z00 | 42057 | Vaginal enterocele NOS |
| K - genitourinary system diseases | K518.00 | 96896 | Female rectocele |
| K - genitourinary system diseases | K51y.00 | 23941 | Other genital prolapse |
| K - genitourinary system diseases | K51yz00 | 41895 | Other genital prolapse NOS |
| K - genitourinary system diseases | K51z.00 | 33440 | Genital prolapse NOS |
| K - genitourinary system diseases | Kyu9100 | 97649 | [x] Other female genital prolapse |
| L - Pregnancy/childbirth/puerperium | L244.11 | 20850 | Cystocele in pregnancy, childbirth and the puerperium |
| L - Pregnancy/childbirth/puerperium | L244.13 | 39492 | Rectocele in pregnancy, childbirth and the puerperium |
| L - Pregnancy/childbirth/puerperium | L244011 | 20907 | Cystocele affecting obstetric care |
| L - Pregnancy/childbirth/puerperium | L244012 | 58517 | Rectocele affecting obstetric care |
| L - Pregnancy/childbirth/puerperium | L244111 | 32286 | Cystocele - baby delivered |
| L - Pregnancy/childbirth/puerperium | L244112 | 32287 | Rectocele - baby delivered |
| L - Pregnancy/childbirth/puerperium | L244211 | 66127 | Cystocele - delivered with postpartum complication |
| L - Pregnancy/childbirth/puerperium | L244212 | 57581 | Rectocele - delivered with postpartum complication |
| L - Pregnancy/childbirth/puerperium | L244311 | 101354 | Cystocele complicating antenatal care - baby not delivered |
| L - Pregnancy/childbirth/puerperium | L244312 | 30378 | Rectocele complicating antenatal care - baby not delivered |
| L - Pregnancy/childbirth/puerperium | L244411 | 38439 | Cystocele complicating postpartum care - baby delivered prev |
| L - Pregnancy/childbirth/puerperium | L244412 | 51111 | Rectocele complicating postpartum care - baby delivered prev |
| L - Pregnancy/childbirth/puerperium | L244z11 | 39550 | Cystocele in pregnancy, childbirth or the puerperium NOS |
| L - Pregnancy/childbirth/puerperium | L244z12 | 64147 | Rectocele in pregnancy, childbirth or the puerperium NOS |
